# A Single Immunization with MVA Expressing GnGc Glycoproteins Promotes Epitope-specific CD8+-T Cell Activation and Protects Immune-competent Mice against a Lethal RVFV Infection

**DOI:** 10.1371/journal.pntd.0002309

**Published:** 2013-07-11

**Authors:** Elena López-Gil, Gema Lorenzo, Esther Hevia, Belén Borrego, Martin Eiden, Martin Groschup, Sarah C. Gilbert, Alejandro Brun

**Affiliations:** 1 Centro de Investigación en Sanidad Animal, INIA, Valdeolmos, Madrid, Spain; 2 Friedrich-Loeffler-Institut, Federal Research Institute for Animal Health, Greifswald-Insel Riems, Germany; 3 Jenner Institute, University of Oxford, Oxford, United Kingdom; Centers for Disease Control and Prevention, United States of America

## Abstract

**Background:**

Rift Valley fever virus (RVFV) is a mosquito-borne pathogen causing an important disease in ruminants often transmitted to humans after epizootic outbreaks in African and Arabian countries. To help combat the spread of the disease, prophylactic measures need to be developed and/or improved.

**Methodology/Principal Findings:**

In this work, we evaluated the immunogenicity and protective efficacy of recombinant plasmid DNA and modified vaccinia virus Ankara (rMVA) vectored vaccines against Rift Valley fever in mice. These recombinant vaccines encoded either of two components of the Rift Valley fever virus: the viral glycoproteins (Gn/Gc) or the nucleoprotein (N). Following lethal challenge with live RVFV, mice immunized with a single dose of the rMVA-Gn/Gc vaccine showed no viraemia or clinical manifestation of disease, but mounted RVFV neutralizing antibodies and glycoprotein specific CD8+ T-cell responses. Neither DNA-Gn/Gc alone nor a heterologous prime-boost immunization schedule (DNA-Gn/Gc followed by rMVAGn/Gc) was better than the single rMVA-Gn/Gc immunization schedule with regards to protective efficacy. However, the rMVA-Gn/Gc vaccine failed to protect IFNAR^−/−^ mice upon lethal RVFV challenge suggesting a role for innate responses in protection against RVFV. Despite induction of high titer antibodies against the RVFV nucleoprotein, the rMVA-N vaccine, whether in homologous or heterologous prime-boost schedules with the corresponding recombinant DNA vaccine, only conferred partial protection to RVFV challenge.

**Conclusions/Significance:**

Given the excellent safety profile of rMVA based vaccines in humans and animals, our data supports further development of rMVA-Gn/Gc as a vaccine strategy that can be used for the prevention of Rift Valley fever in both humans and livestock.

## Author Summary

Rift Valley fever (RVF) is an important disease of ruminants that affects most African and Arabian Peninsula countries where domestic livestock is the basis for subsistence in rural areas. The disease is caused by a bunyavirus that can be transmitted by close contact with infected animals or through the bite of infected mosquitoes thus facilitating the spread of the virus. Safer and practical methods to control virus spread are demanded in order to prevent both human and animal disease after disease outbreaks. The efficacy of a recombinant modified poxvirus vector (the vaccinia modified Ankara virus (rMVA)) and/or DNA-based vaccines in a mouse infection model has been investigated. A single immunization with a rMVA encoding the virus envelope glycoproteins provided sufficient immunity to protect mice against a lethal dose of RVFV. The immune mechanisms underlying the protection were also investigated. A number of specific immune CD8+-T cells could be activated in the presence of at least three different glycoprotein epitopes. On the other hand, the protective effect of the vaccine was found only in immune competent mice since in mice lacking IFN-type-I responses the vaccine was not efficient.

## Introduction

Rift Valley fever virus (RVFV) is a mosquito-borne pathogen causing periodic outbreaks of disease in livestock as well as numerous human infections and fatalities in many African countries (reviewed in [1]). The disease tends to occur following periods of unusually heavy rainfall which favors overgrowth of mosquito populations from trans-ovarially infected eggs [2]. RVFV has the potential to spread to distant geographic regions. After extensive mainland outbreaks [3], [4], [5], [6], [7], [8] the disease has since appeared in the Arabian Peninsula [9] and several Indian Ocean islands [10], [11], [12], [13]. This ability to cross geographical barriers raises concerns of potential spread to RVFV-näive areas [14]. Several promising veterinary livestock vaccines against RVF have been developed [15], [16], [17], the most advanced of which is a live-attenuated vaccine termed ‘Clone 13’ that has been licensed for use in several countries in Africa [18]. However, there is currently no licensed Rift Valley fever vaccine for human use.

Non-replicating, recombinant modified vaccinia virus Ankara (rMVA) has been used widely as a vaccine antigen-delivery platform in previous [19], [20], [21] and numerous ongoing clinical trials against different infectious diseases and cancer [22], [23], [24], [25], [26], [27], [28]. rMVA based vaccines have an excellent safety profile and are proficient inducers of both humoral and cellular immune responses. Poxviruses, including vaccinia virus, are potent inducers of type-I and II interferons and have evolved to encode soluble receptors that may counteract host antiviral mechanisms. Due to deletions in the rMVA genome, the expresfsion of such antagonists is largely absent. This fact contributes to the immunogenicity of rMVA-based vaccines since type-I interferons (IFNα and IFNβ) may act as a link between the innate and adaptive immune system, including antibody and T-cell responses [29], [30].

DNA vaccines encoding both viral RVFV glycoproteins (Gn and Gc) have been tested in Balb/c mice with a varying degree of protection, from full protection without apparent clinical display [31] to intermediate or incomplete protection [32], [33]. In addition, DNA immunization constitutes a safe and efficacious strategy for priming immune responses against a variety of viral pathogens, enhancing immunity of vaccines [34], [35].

In this work we evaluate the immunogenicity and protective efficacy of two rMVA-based vaccines against RVFV in BALB/c mice; one vaccine encodes the RVFV glycoproteins (hereafter termed ‘rMVA-Gn/Gc’) and the other the RVFV nucleoprotein (hereafter termed ‘rMVA-N’). We assess these rMVA vaccines in relation to recombinant DNA vaccines encoding similar RVFV antigens and explore potential mechanisms underlying vaccine-induced immunity using a transgenic IFNAR^−/−^ mouse model. We find that a single immunization with rMVA-Gn/Gc is sufficient for protection against lethal challenge with live RVFV in immunocompetent mice.

## Materials and Methods

### Animals and ethics statement

6 to 8 weeks old BALB/c HSD-Ola H-2K^d^ female mice (Harlan Ltd) or the 129Sv/Ev parental strain and transgenic 129Sv/Ev IFNAR^−/−^ mice (B&K Universal) were used in the experiments described here. All experimental procedures were approved and supervised by the Biosafety and Bioethics Committee from Instituto Nacional de Investigación y Tecnología Agraria y Alimentaria (INIA), following regulatory guidelines from the European Community Council Directive 86/609/EEC.

### Construction of plasmids and generation of rMVA viruses

**Figure 1:**
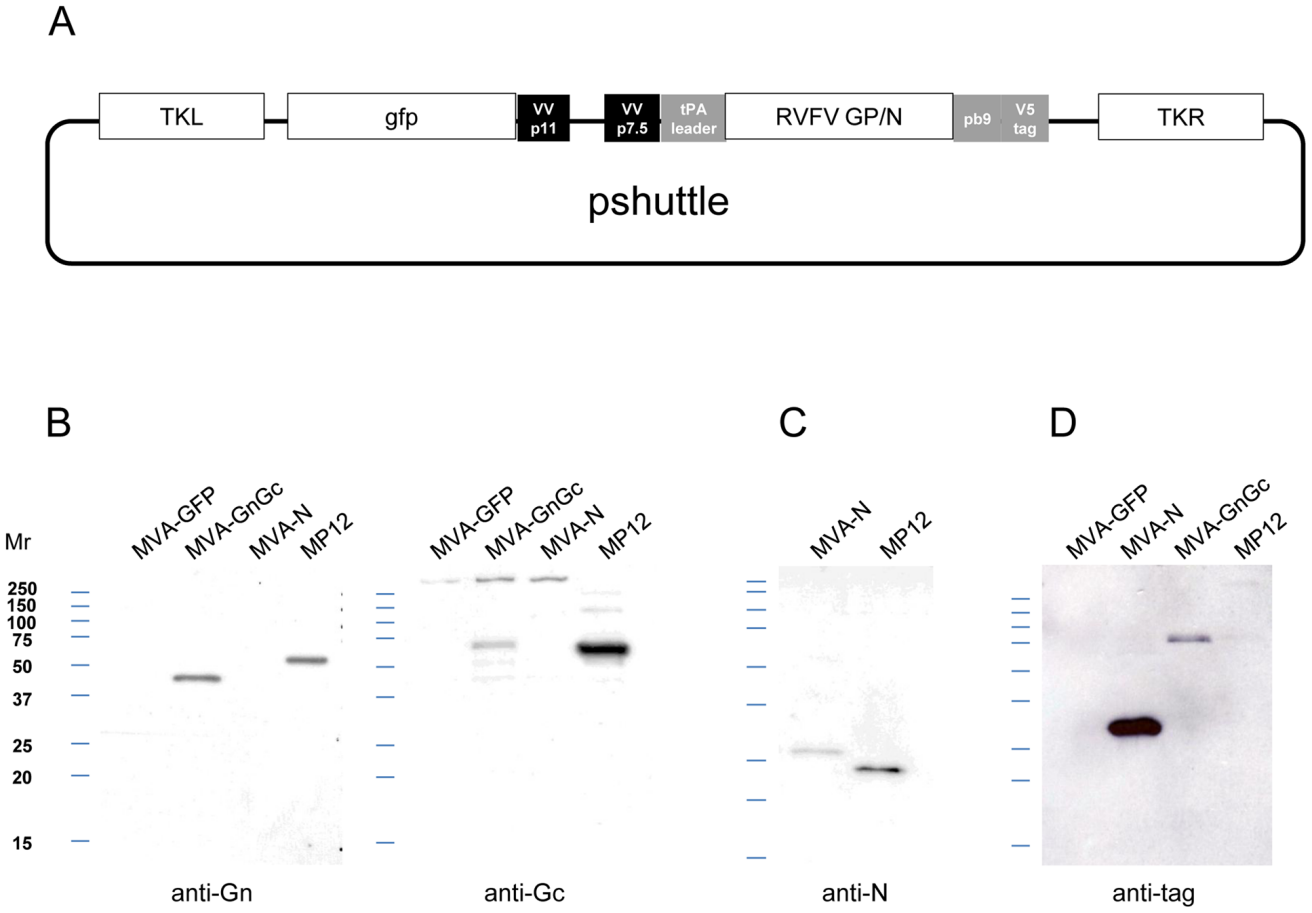
Construct design and analysis of the expression of recombinant MVA viruses. (A) Schematic representation of the plasmid shuttle vector used for the generation of the recombinant rMVA indicating the 5′ and 3′ sequences flanking the RVFV glycoprotein and/or nucleoprotein ORFs. The plasmid derived from pSC11. (B) Expression of Gn and Gc glycoproteins and nucleoprotein N assessed by western blot of rMVA infected BHK21 cell extracts immunoblotted with mAb 84a anti-Gn or a rabbit polyclonal serum anti-Gc. (C) Detection of nucleoprotein N expression in rMVA-N and RVFV (MP12 strain) infected cultures by mAb 2B1. (D) Detection of expressed polypeptides in rMVA-N and rMVA-Gn/Gc infected BHK21 cells using the anti-V5 tag mAb.

Construction of DNA plasmids expressing the RVFV glycoproteins (termed pCMV-M4) and nucleoprotein (pCMV-N) has been described previously [36], [37]. The pCMV vector contains the cytomegalovirus immediate-early promoter (CMV-IE) for initiating transcription of eukaryotic inserts and the SV40 polyadenylation signal (SV40 poly A) for processing the 3′ end of the mRNA transcripts. The rMVA-N and rMVA-Gn/Gc vaccines were constructed by cloning the complete open reading frame of RVFV nucleoprotein from the RVFV MP12 strain using RVFV S-segment cDNA sequence specific primers (GeneBank accession number DQ380154) or the mature Gn/Gc open reading frame (termed M4 and starting at the fourth in frame ATG) using RVFV M-segment cDNA sequence specific primers (GeneBank accession DQ380208), respectively. The RVFV coding sequences were PCR amplified from plasmids pXL-TOPO-N and pXL-TOPO-M1 previously described [36], [37]. The PCR products were ligated to other sequences to create in-frame fusions of the human tissue plasminogen activator (tPA) leader at the N terminus, a T-cell H-2K^d^ restricted epitope (Pb9) from *Plasmodium berghei* and a monoclonal antibody recognition tag at the C terminus (SV5Pk1tag). The inclusion of the tPA leader sequence was based on its association with increased expression and immunogenicity of other genes expressed in the MVA system as previously described [38], [39]. The C-terminal tag can be recognized by specific monoclonal antibodies and consists of the amino acid sequence IPNPLLGLD. The theoretical size for the resulting ORFs was 62.5 kDa for Gn (excluding the tpA leader sequence), 57.4 kDa for Gc (including the pb9 and pk1 tags) and 33.2 kDa for N. The resulting tPA/M4/Pb9/Pk1 and tPA/N/Pb9/Pk1 sequences were then ligated into a shuttle vector, pMVA-GFP, which places the open reading frame under the control of the vaccinia p7.5 early/late promoter, and also includes GFP as a marker gene under the control of the vaccinia p11 late promoter (Figure 1A). The shuttle vector was transfected into chick embryo fibroblasts (CEF) infected with MVA and homologous recombination allowed the shuttle vector to recombine with the MVA genome, inserting the RVFV open reading frames and GFP marker gene at the TK locus of MVA. The recombinant viruses (rMVA-N and rMVA-Gn/Gc) were plaque purified and then expanded in Doug Foster-1 (DF-1) cells.

### Western blot analysis

BHK-21cell extracts, either infected with RVFV, rMVA or transfected with plasmids were subjected to SDS-PAGE in Laemmli's buffer and blotted onto nitrocellulose membranes. Detection of viral proteins was performed using specific anti-Gn monoclonal antibodies (mAb), Gc-specific rabbit antiserum (S. Jäckel et al, unpublished data) or anti-N mAb [40]. Detection of the SV5Pk1 tag epitope was performed with mAb SV5-Pk1 (Serotec).

### Preparation and titration of viral stocks

All animal challenge studies were performed using the South African RVFV isolate 56/74 [41]. The challenge virus stock was propagated twice in BHK-21 cells and titrated by plaque assay in Vero cell monolayers. BALB/c mouse lethal infection dose was estimated by intraperitoneal inoculation of log_10_ dilutions of the stock virus. Preparation of rMVA vaccine stocks was performed by infection of permissive chicken embryo fibroblast (CEF) cells at low m.o.i., the supernatant collected and the remaining cells broken by successive rounds of freezing and thawing. The extracellular virus and released intracellular virus were further concentrated by centrifugation through 36% (w/v) sucrose cushions and titers determined by routine plaque assay in DF-1 cell monolayers.

### Experimental vaccination and RVFV challenge of mice

Groups of 5–7 mice were vaccinated with different vaccine constructs and schedules as summarized in Table 1. 10^7^ plaque-forming units (pfu) of rMVA were administrated intraperitoneally in 200 µl volumes, while 100 µg of plasmid DNA vaccines were administered intramuscularly in 100 µl volumes. All booster doses were administered two weeks after the first vaccination. Fifteen days after the last immunization mice were bled and inoculated i.p. with 10^3^ pfu of the pathogenic RVFV 56/74 strain [41]. This strain had been isolated from a cow after 3 passages in chicken embryo cells and 7 passages in MDBK cells. To monitor viremia, 50–100 µl blood samples were taken at days 3, 6, 10 and 13 days after RVFV challenge. The mice were maintained under observation up to day 23 post challenge. During the course of the experiment all mice were housed in a BSL-3 containment area with food and water supplied *ad libitum*. Clinical signs including ruffled fur, hunched posture, reduced activity or conjunctivitis were monitored for three weeks after RVFV challenge. Viremia was monitored by RT-PCR and virus isolation.

**Table 1:** Morbidity, mortality and antibody responses in BALB/c mice immunized with DNA and/or rMVA vaccines.

Vaccine group	Morbidity^a^	Mortality^b^	Neutralizing antibodies^c^		N-ELISA^d^	
			Prechallenge	Postchallenge	Prechallenge	Postchallenge
**pCMV-M4 (2X)**	5/7 (17)	2/7	1.49±0.48 (7/7)	2.74±0.39	nd	nd
**pCMV-M4 + MVA-Gn/Gc**	2/7 (6)	2/7	0.81±0.94 (3/7)	2.65±0.67	nd	nd
**MVA-Gn/Gc**	0/7 (0)	0/7	1.5±0.05 (7/7)	2.93±0.26	nd	nd
**pCMV-N (2X)**	3/7 (4)	3/7	nd	2.81±0.55	0.96±0.15	nd
**pCMV-N + MVA-N**	6/7 (13)	2/7	nd	2.41±0.27	1.38±0.43	1.87±0.07
**MVA-N (1X)**	6/6 (9)	6/6	nd	-	0.24±0.14	-
**MVA-N (2X)**	5/5 (14)	2/5	nd	nd	1.11±0.05	1.56±0.03
**MVA-Gn/Gc + MVA-N**	1/7 (3)	1/7	1.5±0.05 (4/7)	1.98±0.47	0.72±0.3	1.07±0.27
**Non-vaccinated control**	7/7 (5)	6/7	nd	nd	0.38±0.13	1.76
**MVA control**	6/6 (7)	4/6	0.097 (0/7)	nd	0.18±0.02	2.90*
**pCMV control**	4/4 (5)	4/4	0.097(0/4)	-	0.11±0.04	-

a : Sick mice/total mice. Numbers in parenthesis indicate cumulative days showing clinical display.

b : Dead mice/total mice.

c : Mean ± SD log_10_ titers based on a 50% cytopathic effect reduction; in parenthesis, seroconverted mice/total mice.

d : Mean ± SD ELISA OD_450_ serum titers at 1/80 dilution.

nd: not done.

* only one mouse serum was available for testing.

### Histopathological examination

All dead or culled animals were subjected to histopathological analysis. Liver, spleen, kidney and brain tissue samples were collected and fixed with 10% formaldehyde in phosphate-buffered saline (PBS), embedded in paraffin and sectioned at 4 µm. Hematoxylin and eosin technique was used for histological studies and the avidin-biotin peroxidase complex (ABC) technique used for immunohistochemical detection of specific viral nucleoprotein antigen in liver and brain samples using the anti-nucleoprotein mAb F1D11 [40]. Scores were assigned according to the degree and extent of observed pathology. For liver samples: +3 =  massive destruction of hepatic architecture associated with an intense (+3) nucleoprotein staining; +2 =  severe hepatocellular necrosis with occasional intranuclear bodies accompanied of mixed inflammatory infiltrates; +1 =  moderate hepatic necrosis in discrete areas usually stained with anti-N antibody. For spleen samples pathology was categorized according to varying levels of necrosis found in the germinal centers (GCN), lymphoid cell depletion (LD) and hyperplasia (HP).

### Analysis of peptide specific T-cell stimulation

A selection of class-I restricted H2-K(D)^d^ epitopes on the RVFV Gn, Gc and N primary sequence was performed using available prediction software (immunepitope.org). Peptides were selected on the basis of their highest theoretical affinity score. Peptides with overlapping sequences displaying similar affinity were selected on the basis of their highest predicted proteasomal degradation score. Peptides were synthesized to 95% purity in 5 mg/ml batches by conventional techniques (Proteogenix). The number of IFN-γ secreting T cells in the spleens of naïve, immunized, or RVFV-challenged mice was tested by ELISPOT assay. Briefly, 96-well MAIPS45 Immobilon-P filter plates (Millipore) were coated with 1 µg/ml of anti- IFN-γ capture antibody AG-18/RA-6A2 (BD). Following overnight incubation at 4°C, the wells were washed three times with RPMI 1640 medium and blocked with complete medium supplemented with 10% fetal calf serum for 1 hour at 37°C. 5×10^5^ splenocytes/ml were seeded onto 96-well plates with RPMI medium in the presence of 1 µM of each peptide and incubated for 18 hours at 37°C in the presence of 5% CO_2_. The plates were then washed extensively with distilled water and PBS and incubated with 1 µg/ml of anti- IFN-γ biotinylated mAb R46A2 (BD) for 2 hours at room temperature. Afterwards, plates were washed with PBS and 50 µl of peroxidase-labeled streptavidin at a 1/500 dilution in PBS added to each well and incubated at room temperature for 1 hour. Spots were visualized by the addition of 3-amino-9-ethylcarbazole substrate (BD ELISPOT AEC substrate set) and counted under magnification lens. Control wells with medium alone or with phytohemagglutinin (Sigma Chemical Co.) at a final concentration of 5 µg/ml were also included.

### Quantification of absolute levels and functional activity of IFN-α/β

Levels of biologically active IFN-α/β in serum were determined using a Vesicular Stomatitis virus (VSV Indiana strain) L929 cell cytopathic effect bioassay. L929 cells grown in multi-well plates were incubated for 24 hours with serial 2-fold dilutions of pooled sera from immunized or naïve mice and subsequently infected with 100 TCID_50_ of VSV. Three days after infection the cells were fixed and stained with 10% formaldehyde, 0.3% crystal violet. Serum IFN-α/β titers were expressed as the dilution resulting in a 50% inhibition of infection. Quantification of serum IFN-α was assessed using a commercial mouse IFN-α ELISA (PBL Interferon Source). Known concentrations of mouse interferon were used to generate a standard curve to correlate optical densities with interferon concentration. The sensitivity limit of the assay was estimated in (25 pg/ml)

### Detection of epitope-specific CD8^+^ T-cell responses by ICCS

Mice, either vaccinated with rMVA or plasmid DNA, were sacrificed at day 7 post immunization and their spleens were harvested for analysis by intracellular cytokine staining (ICCS) assay. A total of 10^6^ splenocytes were stimulated with 1 µg/ml of the selected peptides for 6 hours in RPMI 1640 supplemented with 10% fetal calf serum and containing brefeldin A (5 µg/ml) to increase the accumulation of IFN-γ in the responding cells. After stimulation, cells were washed, stained for the surface markers, fixed, permeabilized and stained intracellularly using appropriate fluorochromes. To analyze the adaptive immune responses, the following fluorochrome-conjugated antibodies were used: CD4-FITC, CD8-PerCP and IFNγ-PE. All antibodies were from BD Biosciences. Data were acquired by FACS analysis on a FACSscalibur (Becton Dickinson) and were analyzed with FlowJo (Tree Star 7.6.5 software).

### RVFV neutralization assay

Sera from vaccinated mice were heat-inactivated at 56°C for 30 minutes and serially diluted (two-fold or three-fold, starting a dilution of 1∶20 in DMEM medium containing 2% fetal bovine serum, mixed with an equal volume (50 µl) of medium containing 4×10^3^ pfu of a viral stock (RVFV-MP12 strain). After one hour of incubation at 37°C, this mixture was added to Vero cell monolayers seeded in 96-well plates. After 3 days at 37°C in the presence of 5% CO_2_, cells were fixed and stained with a solution containing 2% crystal violet in 10% formaldehyde. The neutralization titer of each serum sample was defined as the reciprocal of the highest dilution of serum where a 50% of neutralization was observed relative to controls.

### Detection of RVFV antibodies

RVFV nucleoprotein specific antibodies were detected by ELISA as described previously [37]. Briefly, viral antigen from RVFV strain MP12 infected cell cultures was added to 96-well plates pre-coated with rabbit anti-RVFV hyperimmune serum. This serum captures efficiently RVFV nucleoprotein antigen, but not RVFV glycoproteins, at the dilutions used in ELISA. Serial dilutions of sera from immunized mice were added to wells and the immuno-complexes detected with anti-mouse-IgG-HRPO labeled antibody. Bound conjugate was detected using TMB (3,3′,5,5′-tetramethyl-benzidine) Liquid Substrate, Supersensitive (Sigma) for 10 min, followed by one volume of stopping solution (3N H_2_SO_4_). Optical densities were measured at 450 nm (OD_450_). Cut-off ELISA was set to two-fold the OD_450_ value of a pre immune pooled mouse sera.

### Statistical analysis

The log rank (Mantel-Cox) test was used for survival analysis following RVFV challenge. Individual ELISPOT values were determined by subtracting background values obtained after stimulation with media only and log_10_ transformed for analysis. Data from each vaccination group were analyzed using a randomized block analysis of variance. Dunn's and Tukey's post hoc tests were used for multiple comparisons among groups. All analysis was done using the GraphPad 5.0 software (San Diego, CA). Differences were significant when p values <0.05.

## Results

### Expression of RVFV antigens in rMVA infected cells

The expression of recombinant RVFV antigens was assessed by western blot of infected BHK-21 cell extracts using antibodies specific for RVFV glycoproteins Gn and Gc, the RVFV nucleoprotein N and the SV5 tag (Figure 1B). Both rMVA viruses expressed RVFV N and Gc polypeptides of a slightly higher size than those of MP12, according to their theoretical mass. The high intensity of the anti-V5tag immunoblotting signal suggested that the RVFV nucleoprotein N was expressed more efficiently than glycoprotein Gc (Figure 1D). In contrast, the Gn glycoprotein was also detected but at a lower molecular mass than the one expressed in BHK-21 cell extracts infected with RVFV-MP12, potentially indicating differences in post-translational processing of the Gn glycoprotein in the rMVA-Gn/Gc vaccine construct as compared to wild type RVFV.

### Assessment of vaccine immunogenicity and efficacy in BALB/c mice

We assessed the immunogenicity and protective efficacy of several DNA and rMVA vaccination schedules, summarized in Table 1, following challenge with 10^3^ pfu of live RVFV strain 56/74, a dose that ensures close to 100% mortality in BALB/c mice. First, we evaluated vaccine constructs encoding the Gn/Gc viral glycoproteins in three groups of 7 mice each: one group was immunized with rMVA-Gn/Gc only, one with the recombinant DNA vaccine pCMV-M4, and one immunized with both vaccines in a heterologous prime-boost regimen (that is, pCMV-M4 followed by rMVA-Gn/Gc). Groups of unvaccinated mice (n = 7), vaccinated with empty MVA (n = 6) or with empty pCMV plasmid (n = 4) were used as controls.

**Figure 2:**
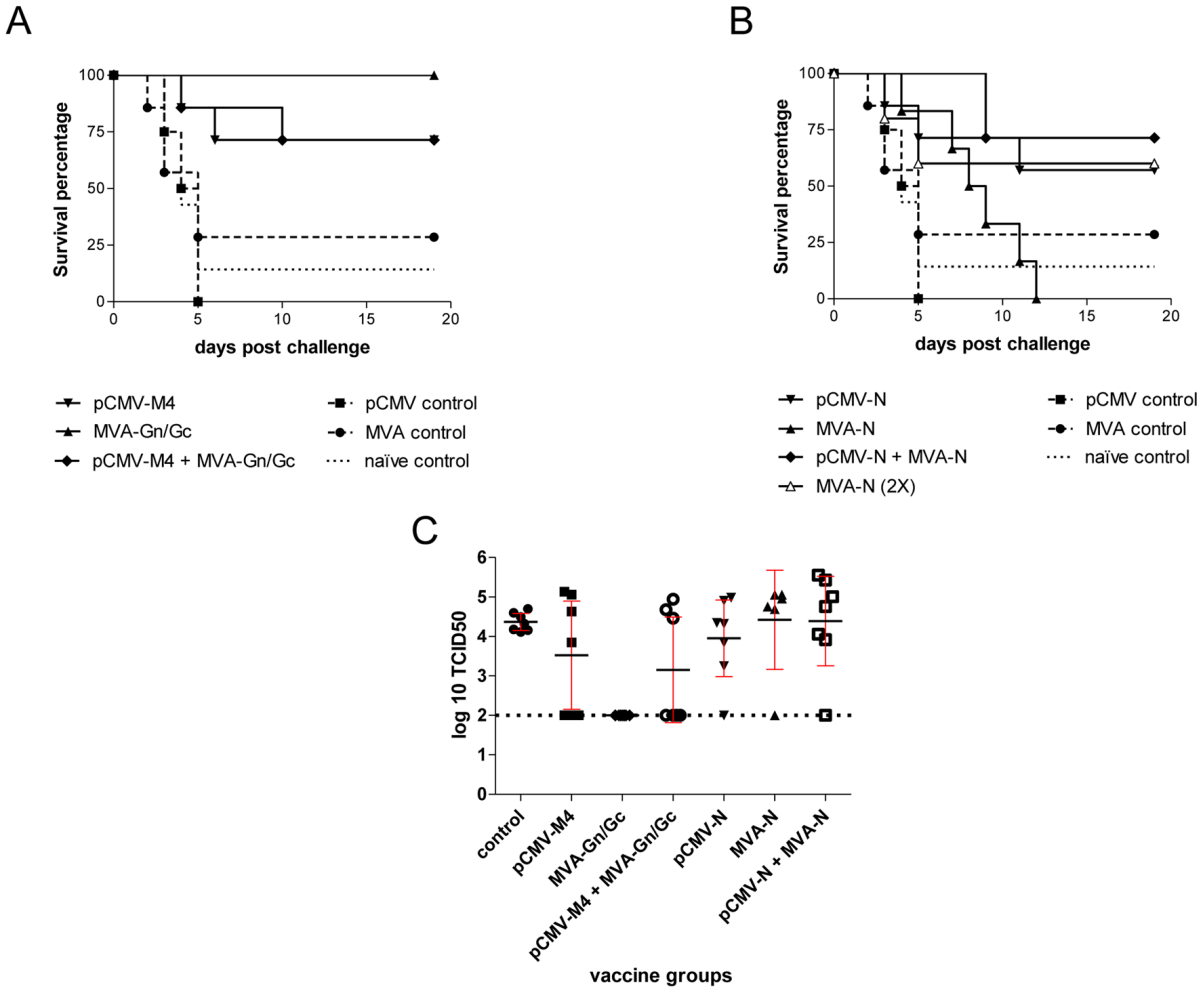
Survival curves and viremia upon RVFV lethal challenge in vaccinated mice. Survival curves of mice vaccinated with glycoprotein (A) or nucleoprotein (B) based-DNA or MVA constructs upon RVFV challenge. (C) Scatter dot representation of viremia titers of individual mice from each vaccine groups measured 3 days post viral challenge. Mean ±95% confidence intervals are shown. The limit of detection of the assay is indicated by the dotted line.

Mice immunized with a single dose of the rMVA-Gn/Gc vaccine mounted a modest RVFV neutralizing antibody response and were fully protected against clinical disease and viremia (Table 1 and Figure 2A). The RVFV neutralizing antibody titers observed in the pCMV-M4-only group were comparable to those induced by the single dose rMVA-Gn/Gc regimen. Despite this, five of the seven mice in the pCMV-M4-only group displayed clinical signs upon RVFV challenge (ruffled fur, hunched-like posture and hypothermia), with two dying at 4 and 6 days post-challenge, respectively.

Among seven mice included in the heterologous pCMV-M4-rMVA GnGc vaccination regimen only two displayed clinical signs of illness, dying shortly afterwards in spite of detectable RVFV neutralizing antibodies in their serum. Four of the five surviving mice had no detectable RVFV neutralizing antibodies (Table 1). However, survival analysis revealed no statistically significant difference between mice with and those without detectable RVFV neutralizing antibodies (Figure 2A).

Next, we evaluated immunogenicity and efficacy of vaccine constructs encoding the RVFV nucleoprotein in five groups of mice: one group (n = 6) was immunized with a single dose of rMVA-N, one group (n = 5) with two doses of rMVA-N, one group (n = 7) with two doses of pCMV-N, one group (n = 7) immunized with both vaccines in a heterologous prime-boost regimen (that is, pCMV-N followed by rMVA-N) and a final group (n = 7) immunized with rMVA-Gn/Gc and rMVA-N in a prime-boost regimen. Groups of unvaccinated mice and immunized with vectors with no insert were used as controls (Table 1).

With the exception of mice vaccinated with a single rMVA-N dose, all mice vaccinated with a nucleoprotein-based vaccine, whether vectored by rMVA or DNA, mounted an antibody response against the RVFV nucleoprotein as detected by ELISA (Table 1). Though anti-N antibodies are not neutralizing, partial protection in experimental mouse models has been consistently observed following immunization targeted at the RVFV nucleoprotein [32], [33], [36]. In keeping with these previous studies, all but the single dose rMVA-N regimen conferred partial protection to RVFV challenge (Table 1 and Figure 2B). The mean viremia titers were comparable across groups (Figure 2C), though the onset and duration of clinical signs of illness differed markedly among the groups. Two mice from the homologous prime-boost pCMV-N group displayed clinical signs as early as 3 days post-challenge with a third mouse displaying clinical signs at 10 days post-challenge. Onset of clinical signs in the group receiving the heterologous prime-boost regimen (that is, pCMV-N followed by rMVA-N) was observed as from 7 days post-challenge. Mice in the homologous prime-boost rMVA-N group also exhibited a delay in onset of clinical signs relative to unvaccinated controls. However, mice in the single dose rMVA-N group, which also lacked detectable anti-N antibodies, showed clinical signs as from 4 days post-challenge and all succumbed to the infection (Table 1 and Figure 2B). Finally, only one of seven mice immunized with both the rMVA-GnGc and rMVA-N vaccines displayed clinical signs of illness (Table 1). All sera from convalescent mice, whether immunized with Gn/Gc or N-based vaccines, had increased neutralizing titers indicative of anamnestic responses (Table 1).

Together, these data suggest that immunization with rMVA-Gn/Gc alone is sufficient to induce full protection in BALB/c mice upon a lethal RVFV challenge. Nevertheless, the different onset times and outcomes in morbidity observed among the various Gn/Gc and N-based vaccination regimens tested suggests that the mechanism underlying vaccine-induced immunity to RVFV may not be solely attributable to the induction of RVFV neutralizing antibodies. Since T-cells would be expected to play a major role in viral clearance we next assessed the extent to which induction of T-cell responses varied among vaccinated mice.

### Analysis of T-cell responses

Several 9-mer peptides derived from Gn/Gc and N primary sequence and predicted to be class-I MHC restricted (Tables 2 and 3) were used in an *ex vivo* IFN-γ ELISPOT assay (see Materials and Methods). The Gn peptide #4 (SYAHHRTLL) was previously identified as MHC-I restricted [42]. On the other hand, peptides #13 (SYKPMIDQL) and #14 (GGPLKTILL), included here, are the first MHC-I restricted epitopes identified to date on the Gc sequence. As a positive control, the nucleotide sequence from the well-characterized CTL epitope pb9 (SYIPSAEKI) from *Plasmodium berghei* [43] was included in frame at the 3′end of both the RVFV nucleoprotein and glycoprotein open reading frames as shown in Figure 1A (also see Materials and Methods). To check if any of the predicted peptides were able to stimulate CD8+-T cells, two mice were inoculated intraperitoneally with one dose of 10^7^ pfu of either rMVA-N or rMVA-Gn/Gc. Splenocytes harvested at 7 days post-immunization were stimulated for 18 hours with 5 µg/ml of each peptide.

**Table 2:** List of predicted class-I-restricted GnGc ORF peptides used in the study.

Peptide #	9-mer sequence	Position in ORF	H-2 haplotype(s) (predicted)
1	LYRALKAII	73–81	K^d^
2	PPHKKRVGI	106–114	D^d^
3	TYAGACSSF	183–191	K^d^
4	SYAHHRTLL	205–213	K^d^
5	CSHANGSGI	434–442	K^d^, D^b^
6	LVLGNPAPI	658–666	D^b^
7	SYASACSEL	686–694	K^d^
8	CGGWGCGCF	818–826	D^b^, D^d^
9	CFNVNPSCL	825–833	D^b^
10	SCLFVHTYL	831–839	K^d^
11	SGSNSFSFI	898–406	K^d^
12	ESPGKGYAI	907–915	D^d^
13	SYKPMIDQL	955–963	K^d^
14	GGPLKTILL	1154–1162	D^d^
15	LYVALSIGL	1165–1173	K^d^

**Table 3:** List of predicted class-I-restricted nucleoprotein ORF peptides used in the study.

Peptide #	9-mer sequence	Position in ORF	H-2 haplotype(s) (predicted)
1	NYQELAIQF	3–11	K^d^
2	AQAVDRNEI	13–21	K^d^, D^b^
3	RGNKPRRMM	64–72	D^d^
4	EGKATVEAL	78–86	D^d^
5	EALINKYKL	84–92	D^b^
6	EGNPSRDEL	94–102	D^d^
7	WLPVTGTTM	125–133	D^d^, D^b^
8	SFAGMDVPS	148–156	K^d^
9	YLLQFSRVI	172–180	K^d^, D^b^
10	FTQPMNAAV	196–204	K^d^
11	NAAVNSNFI	201–210	D^b^
12	SHEKRREFL	210–218	D^d^

**Figure 3:**
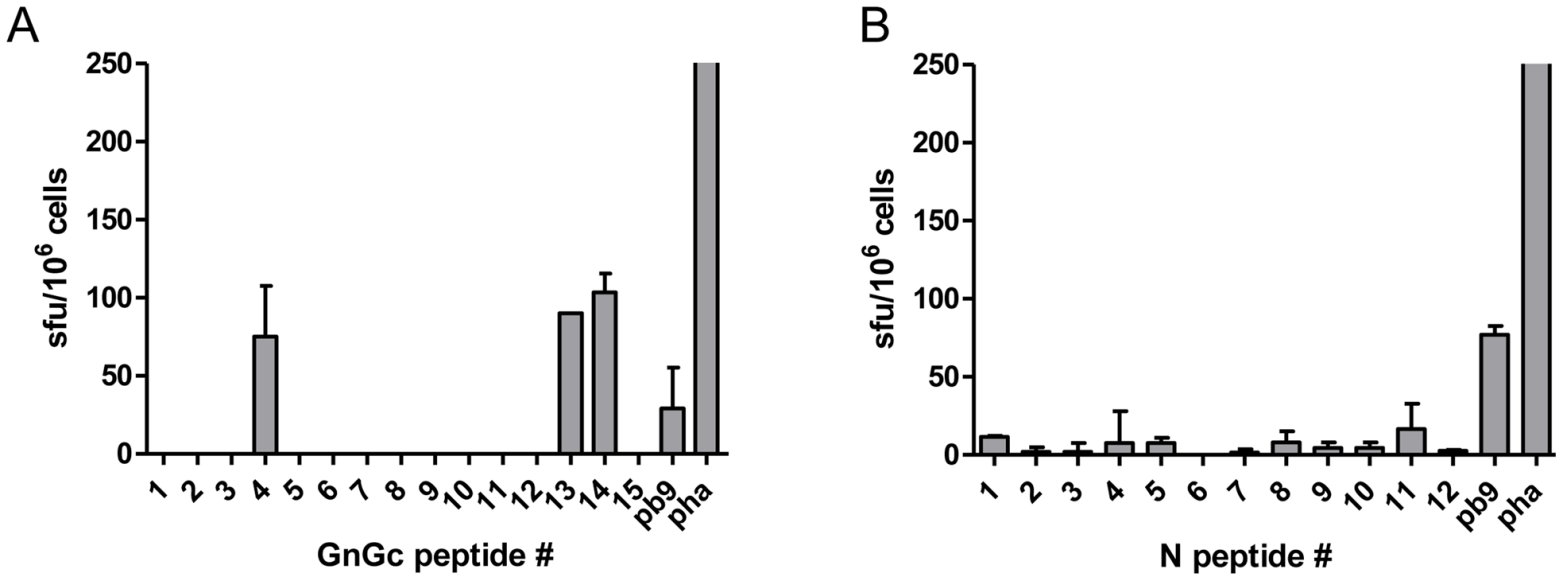
Detection of peptide-specific T-cell responses by interferon-γ ELISPOT in rMVA vaccinated mice. Cellular immune responses in mice immunized with rMVA-N (A) or rMVA-Gn/Gc (B) measured by ELISPOT upon individual peptide stimulation of splenocytes. Values represent mean ± standard deviation (SD) of spot forming units (SFU) using spleen cells from two mice immunized with a single intraperitoneal dose of each rMVA. Peptides are numbered sequentially according to their position on their respective ORFs (see Tables 2 and 3). Data were transformed by subtraction of background (medium only) values obtained with no peptide incubation. Phytohemagglutinin (PHA) was used as a non-specific positive control for cell cytokine expression. A peptide corresponding to the pb9 T-cell epitope was used as recombinant antigen-specific positive control.

Both mice immunized with the rMVA-Gn/Gc vaccine displayed IFN-γ secreting cells in response to peptides #4, #13 and #14 (Figure 3A). Negligible responses were found for the rest of the assayed Gn/Gc peptides. Similarly, none of the epitopes selected from the RVFV nucleoprotein were able to induce IFN-γ secretion upon stimulation, in spite of the clear response induced by the pb9 control peptide, indicating that the rMVA expressed N protein was also efficiently processed through the class-I pathway for antigen presentation (Figure 3B).

**Figure 4:**
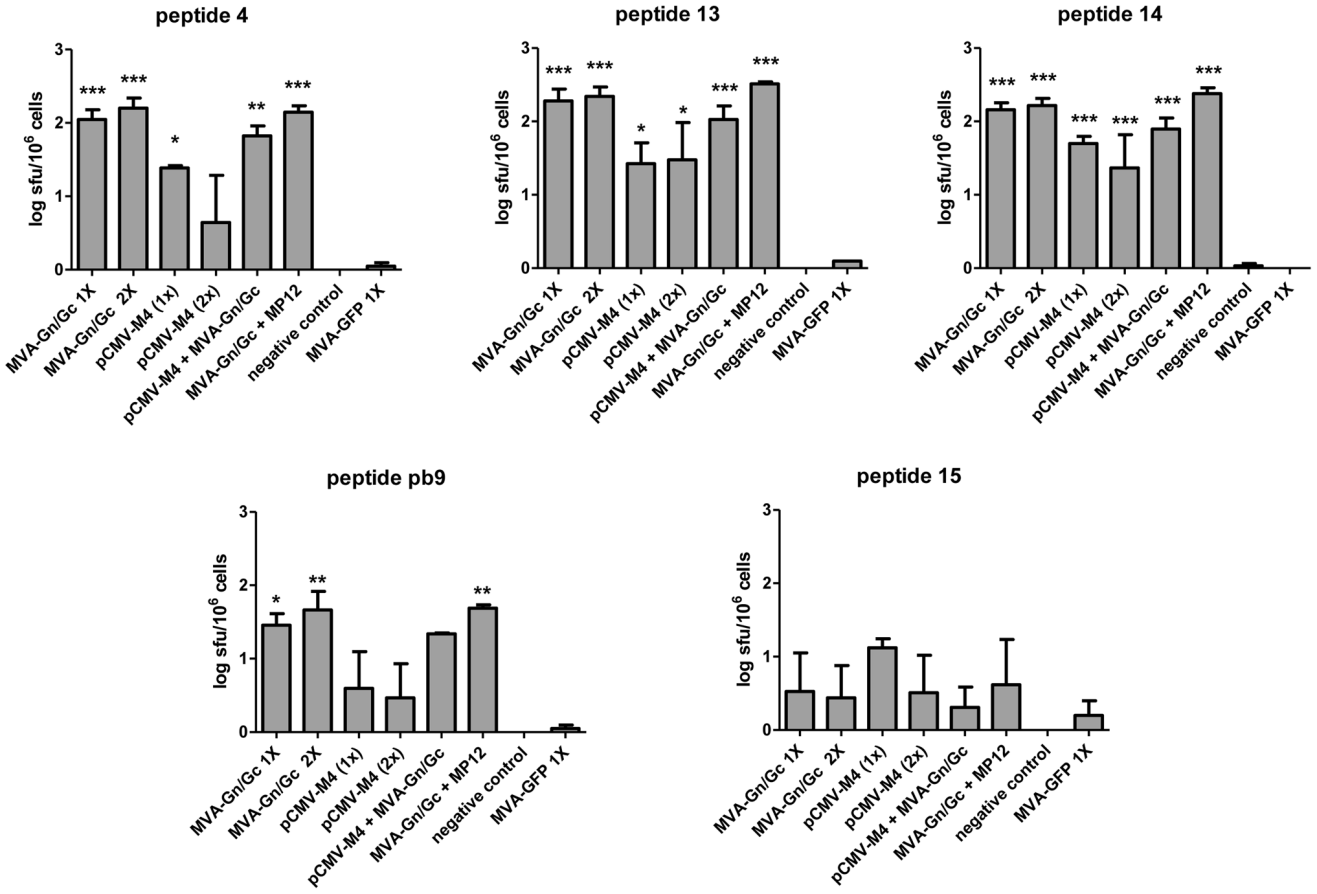
Cellular response against glycoprotein peptides in mice upon different vaccine combinations. Mean ± SD log SFU values obtained in spleen cells from mice immunized with different vaccine combinations. Naïve, non-immunized mice were used as negative controls. Peptides 4, 13 and 14 were selected on the basis of their ability to stimulate Gn and Gc specific T-cell responses. Peptide 15 was used as a non-stimulator peptide to measure the background of the assay. The pb9 peptide was used as a specific positive control for rMVA vaccinated mice. The plots show data from three independent experiments. Statistical significance was calculated by one-way analysis of variance (ANOVA) transforming ELISPOT counts to log_10_ to limit the range of variation found among individual mice. Asterisks indicate the level of significance of each group when compared to the MVA-GFP control group using Dunn's post hoc test (* = p<0.05; ** = p<0.001; *** = p<0.0001).

We next tested whether the observed RVFV glycoprotein-specific peptides were able to induce also the activation of T-cell responses in mice immunized with plasmid DNA alone or in combination with MVA. As shown in Figure 4, the mice vaccinated with either a single or a booster dose of rMVA-GnGc induced consistent T-cell responses in the ELISPOT assay. Mice that were boosted intraperitoneally with 10^4^ pfu of RVFV-MP12 15 days post rMVA GnGc vaccination showed also similar T-cell responses. Mice that were immunized with a single dose of pCMV-M4 also mounted T-cell responses against peptides #4 and #13, albeit lower that the levels induced by the single rMVA-Gn/Gc vaccination. Nevertheless, these responses were clearly increased following a booster dose of rMVA-Gn/Gc to the pCMV-M4 group.

### Identification of CD8^+^ T-cells

**Figure 5:**
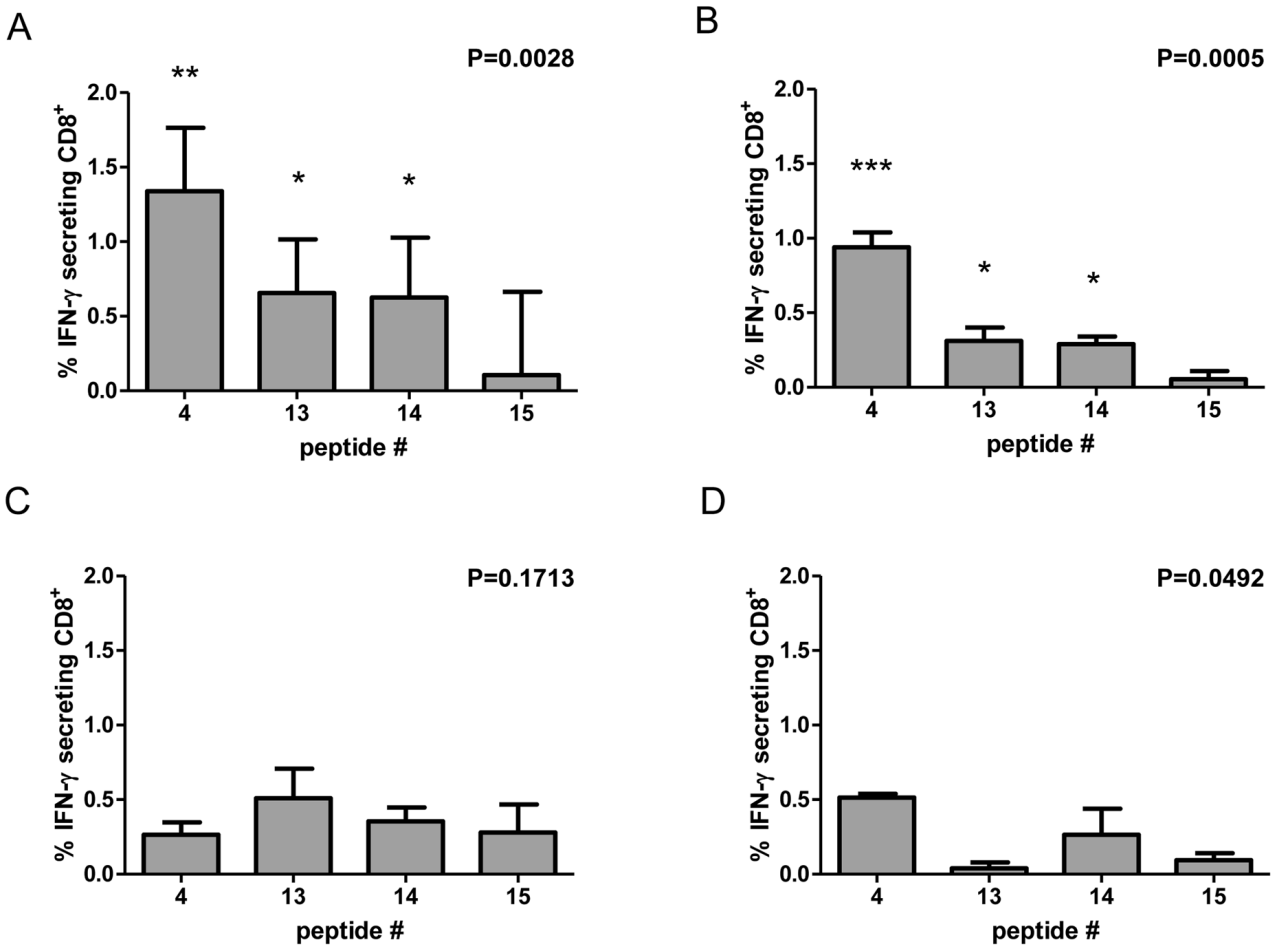
ICCS quantification of CD8+T cells stimulated by glycoprotein-specific peptides. The graphs depict percentages of interferon secreting CD8+T cells (mean ± SD from two mice) of Balb/c mice vaccinated with rMVA-Gn/Gc single dose (A), rMVA-Gn/Gc prime and boost (B), DNA (pCMV-GnGc (M4)) prime and boost (C) and DNA prime and rMVA-Gn/Gc boost (D). One-way ANOVA p values are shown for each group to indicate significant group variation (p<0.05). Peptide #15 was included as a negative (non stimulator) control. Asterisks indicate the level of significance of each group when compared to control peptide #15 with Tukey's post hoc test (* = p<0.05; ** = p<0.001; *** = p<0.0001).

In order to confirm whether the class-I restricted peptides stimulating ELISPOT T-cell responses were indeed recognized by CD8+ T cells, an intracellular cytokine staining assay (ICCS) was performed. Among mice vaccinated once with rMVAGn/Gc a significant percentage of the whole CD8+ T cell population expressed IFN-γ in response to peptides #4, #13 and #14 but not in the presence of the non-stimulator peptide #15 (Figure 5A). This percentage was not increased in mice receiving a booster dose of rMVAGn/Gc (Figure 5B). While DNA vaccination (either single or two dose administration) was not able to trigger significant glycoprotein specific CD8+T cell stimulation (Figure 5C), the heterologous prime-boost (pCMV-M4 followed by rMVA-Gn/Gc) was sufficient to trigger CD8+ T cell stimulation (Figure 5D), although this was restricted to peptides #4 and #14 and at lower levels than those observed for rMVA-Gn/Gc immunization alone or with a booster dose. Strikingly, a booster dose of RVFV MP12 strain in mice immunized with rMVA-Gn/Gc did not suffice to stimulate Gn or Gc peptide-specific CD8+T cells (data not shown), in spite of the T-cell responses observed with this vaccine regime in the ELISPOT assay. Presence of peptide stimulated IFN-γ secreting CD4+ T cells were also analyzed but no detectable staining was achieved (not shown).

### Role of type I IFN response in the protective capacity of rMVA vaccines

**Figure 6:**
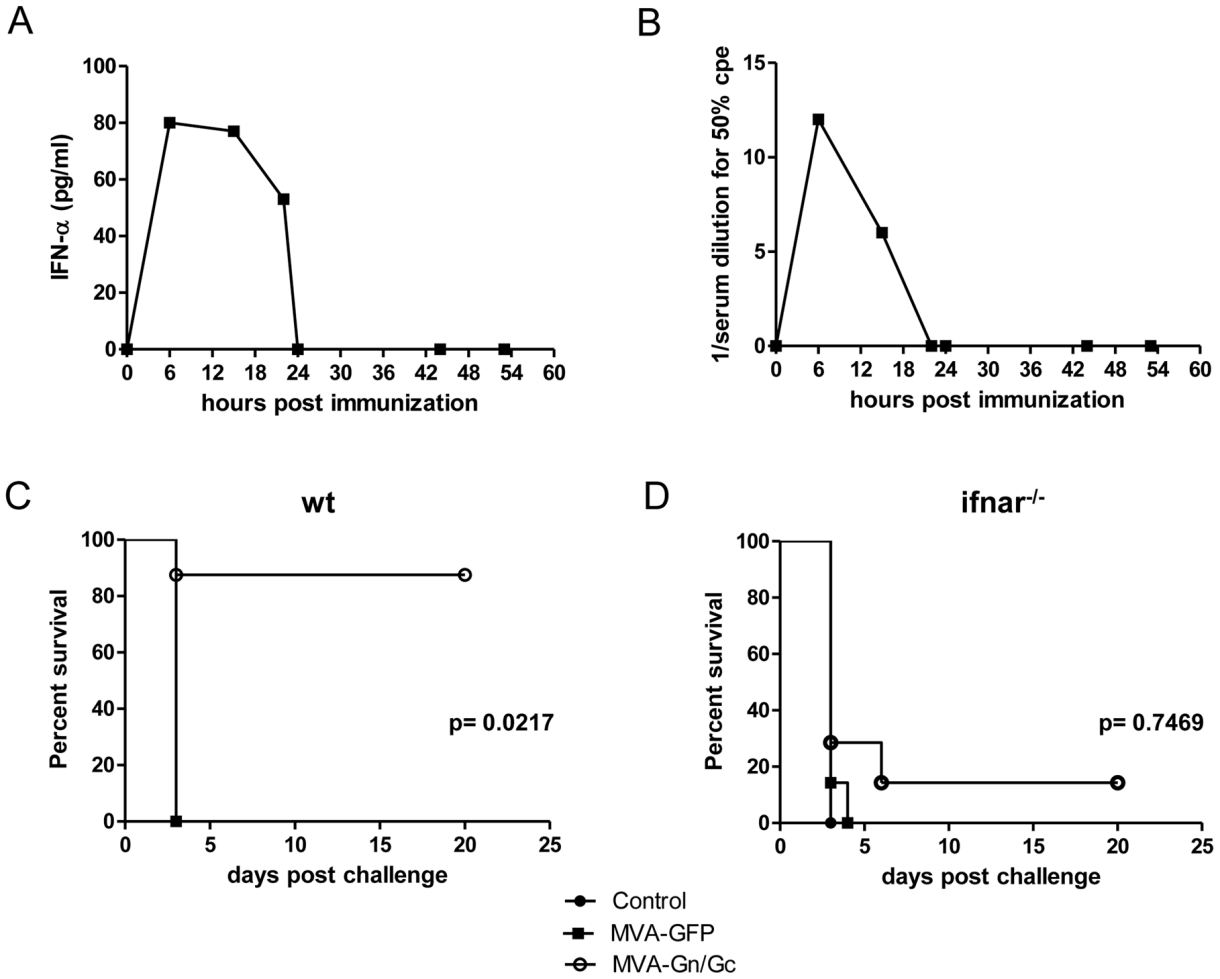
Role of functional type I IFN response in the protection elicited by rMVA-Gn/Gc. (A) Detection of IFN-α in 129Sv/Ev mice vaccinated with a single dose of rMVA-Gn/Gc vaccine. Kinetics of ELISA IFN-α levels (A) and antiviral activity (B) in pooled sera from immunized mice. (B) Survival curves upon RVFV lethal challenge in vaccinated 129Sv/Ev wt (C) or IFNAR^−/−^ mice (D). The mice were vaccinated with a single dose administration and challenged as described. Statistical significance was considered when p<0.05 as determined by the log rank (Mantel-Cox) test.

Since the rMVA virus has lost several genes encoding soluble receptors, it is unable to efficiently counteract host's innate immune responses. Therefore, upon rMVA infection the levels of systemic IFN-α/β should raise temporarily allowing a window for cytokine detection. To test whether immunization of rMVA-Gn/Gc was indeed inducing the expression of these cytokines, sera from mice were taken at different times post-immunization and tested for the presence of IFN-α. An increase in the level of IFN-α was clearly detected, peaking at 6 hours post-immunization and spanning for at least for 24 hours (Figure 6A, left). We also tested whether the serum levels of this cytokine were biologically active to prevent propagation of an interferon sensitive virus such as Vesicular stomatitis virus (VSV). A peak of antiviral activity was observed around 6 hours post immunization, decreasing afterwards (Figure 6A, right). Since the timing of IFN-α detection was coincidental, the observed antiviral activity could be attributed at least in part to the effect of IFN- α.

Since IFN-α/β modulates the induction of subsequent adaptive responses, including CD8+T cell specific immune responses, we also tested the role of these cytokines in the protection elicited by the rMVA-Gn/Gc vaccine. 129Sv/Ev IFNAR^−/−^ mice and 129Sv/Ev wild type mice were immunized intraperitoneally with 10^7^ pfu of rMVA-Gn/Gc vaccine and challenged with a lethal dose of RVFV 56/74. The level of prechallenge neutralizing antibody titers ranged between 1/20 and 1/40 for both groups of mice respectively, similar to those obtained in the BALB/c model. While 8 out of 9 wild-type 129 mice survived the challenge, only one out of 7 IFNAR^−/−^ mice survived the challenge (Figure 6B). Moreover, the protective effect was glycoprotein-specific and not solely due to the IFN type I induction since 129Sv/Ev wild type mice vaccinated with a control rMVA expressing an irrelevant antigen (GFP) did not survive the challenge. These data emphasize the important role of the innate immune response in the shaping of subsequent adaptive responses.

### Pathological findings

Most mice used in the challenge study were subjected to pathological examination. Mice could be classified into three distinct groups based on pathological findings and independent of vaccination regimen (Table 4). First, all mice dying before 8 dpi showed pathology indicative of an acute onset hepatitis with intense viral replication in liver characterized by massive hepatic necrosis that correlated with intense staining by immunohistochemistry using anti-N mAb F1D11 [40]. Splenic lymphoid depletion of varying severity, including the presence of necrotic cells and cellular debris in germinal centers, was also consistently observed in these mice.

**Table 4:** Summary of the pathological findings in challenged mice.

Days to death	Mouse ID	Vaccine group	HN	LD	GCN	HP	TN	BG	N antigen (liver)
**3**	**6**	control	+++	++					+
**3**	**5**	control	+++	+++	++		++		++
**3**	**2**	control	+++	++++	++				++
**4**	**5**	DNA GnGc (2X)	+++	++	+++				++
**4**	**4**	control	+++	++++	++				+++
**4**	**2**	MVA N (1X)	+++	++++	+++				+++
**4**	**5**	DNA GnGc +MVA GnGc	+++	+++	+++				+++
**3**	**5**	DNA N (2X)	+++	++	++				+++
**5**	**7**	control	+++	++++	+++				+++
**5**	**1**	control	+++	++++	+++				+++
**5**	**4**	DNA N (2X)	+++	++++	+++				+++
**6**	**6**	DNA GnGc (2X)	+++	++	++				+
**7**	**1**	MVA N (1X)	nd	nd	nd				nd
**8**	**5**	MVA N (1X)	+++	+++	+++				+++
**9**	**7**	DNA N +MVA N		++	++				−
**9**	**4**	DNA N +MVA N	nd	nd	nd				+/−
**9**	**4**	MVA N (1X)	+			++			−
**10**	**3**	DNA GnGc +MVA GnGc		++++					−
**10**	**6**	MVA GnGc + MVA N				+			+/−
**11**	**7**	DNA N (2X)		++					−
**11**	**3**	MVA N (1X)		++++	++				−
**12**	**6**	MVA N (1X)		++	++				−
**19**	**3**	DNA GnGc (2X)		++					−
**19**	**7**	DNA GnGc (2X)		++			++		−
**19**	**4**	DNA GnGc (2X)		++++	++++				−
**19**	**2**	DNA GnGc (2X)		+					−
**19**	**1**	DNA GnGc (2X)		++++			++	++	−
**19**	**2**	DNA N (2X)		+					−
**19**	**2**	DNA GnGc +MVA GnGc							−
**19**	**1**	DNA N +MVA N	+			+		++*	−
**19**	**6**	DNA N +MVA N		+					−
**19**	**2**	DNA N +MVA N							−
**19**	**5**	DNA N +MVA N				+			−
**19**	**3**	DNA N +MVA N							−
**19**	**1**	MVA GnGc		+			+		−
**19**	**4**	MVA GnGc		++					−
**19**	**2**	MVA GnGc + MVA N		++					−
**19**	**3**	control		++				++	−
**24**	**7**	DNA GnGc +MVA GnGc		+++					+/−
**24**	**1**	DNA GnGc +MVA GnGc		+++					−
**24**	**4**	DNA GnGc +MVA GnGc		+++					−
**24**	**6**	DNA GnGc +MVA GnGc		+					−
**24**	**5**	MVA GnGc		+					+/−
**24**	**2**	MVA GnGc		+					−
**24**	**6**	MVA GnGc							−
**24**	**7**	MVA GnGc		++					−
**24**	**3**	MVA GnGc		++					−
**24**	**3**	DNA N (2X)		++					−
**24**	**6**	DNA N (2X)							−
**24**	**1**	DNA N (2X)		+					−
**25**	**7**	MVA GnGc + MVA N							−
**25**	**1**	MVA GnGc + MVA N							−
**25**	**3**	MVA GnGc + MVA N							−
**25**	**5**	MVA GnGc + MVA N							−
**25**	**4**	MVA GnGc + MVA N		+					−

HN = hepatic necrosis; LD = Spleen lymphoid depletion; GCN = necrosis in germinal centers; HP = splenic hyperplasia; TN = tubular necrosis in kidney; BG = brain gliosis (*associated with viral antigen). Liver pathology was categorized from massive (+++) to moderate (+). Spleen pathology was ranked as severe (++++), intense (+++), moderate (++) or mild (+). Immunohistochemistry for viral N antigen detection; intense (+++), moderate (++), mild (+), weak (+/−).

Second, a group of mice that survived longer (9 to 12 days post challenge) did not show acute hepatitis but had affected spleen areas with different degrees of lymphoid depletion. The cause of death of these mice remains unclear since most of the mice that survived the challenge also displayed similar spleen alterations although in a few cases brain areas showed gliosis as a distinct feature. Finally a third group of surviving mice either did not show any particular pathology or very mild affectation was observed in spleen in the form of lymphoid depletion. Two of these mice showed very mild viral antigen staining in the liver and other three mice displayed gliosis in brain, in one of them it was found associated to viral nucleoprotein staining.

## Discussion

Previous reports have shown that DNA plasmid constructs encoding mature RVFV glycoproteins (Gn/Gc) protected BALB/c mice from a virulent RVFV challenge, showing full protection without apparent clinical signs [31]. In contrast, the protection achieved in our study was not complete though close to 75%. Nevertheless, our data are consistent with the ability of DNA vaccines encoding RVFV glycoproteins to confer protection in mice after a virulent RVFV challenge, although we did observe clear clinical display in BALB/c mice. These differences in mortality and morbidity with previous studies could be explained by the lower number of immunizations (two vs three) and/or the i.m saline delivery method used for vaccination, perhaps less efficient than gene gun delivery in terms of eliciting protective immune responses (saline-DNA immunization raises a predominantly Th1 response with mostly IgG2a antibodies, while gene gun DNA immunization produces a predominantly Th2 response with mostly IgG1 [44]). This is also true for DNA immunization using RVFV glycoprotein Gn as immunogen [42]. Our initial aim was to improve the efficiency of a DNA vaccine approach by testing the option of using plasmid DNA encoding both RVFV glycoproteins to prime immune responses following by a booster dose with a recombinant MVA vector expressing the same antigen. Interestingly, MVA expressed Gn showed differences in size compared to that of MP12 that could not be attributed to differential glycosylation, cell type or MVA encoded proteolytic activities (data not shown). Therefore, further work will be required to establish the molecular basis for this difference. DNA/MVA heterologous prime-boost strategy has been shown to enhance the levels of T-cell responses for some intracellular pathogens [45], [46]. In our hands a combined heterologous DNA prime-rMVA-Gn/Gc boost reduced the previously observed morbidity in DNA-only vaccinated mice after a lethal challenge, although titers of neutralizing antibodies were not consistently raised in all mice (as those achieved with two serially administered plasmid doses). Moreover, some of the mice in which no neutralizing antibodies were detected were able to survive the challenge. Although the level of neutralizing antibodies detected was rather low, the lack of a good correlation between protection and induction of neutralizing antibodies was suggestive of other immune mechanisms responsible for the level of protection afforded. Though the overall protection achieved by the DNA-only or prime-boost approaches was higher that the observed in control groups, statistical significance (p≤0.05) was not reached when compared with the MVA control group (log-rank test p = 0.0889; df = 2; χ^2^ = 4.841) On the other hand, the observation that a single ip administration of rMVA-Gn/Gc achieved higher protection levels than a DNA/MVA prime boost indicated that the immune consequences of both vaccines were nor additive nor synergistic when triggering glycoprotein-specific protective immune responses. In contrast, an enhancement of anti-N antibody responses was achieved by DNA/MVA prime boost when compared to vaccination with DNA or MVA-N alone. Perhaps the nature, subcellular location and synthesis of the vaccine antigen and delivery method used greatly influences the outcome of immune responses. The mechanism of protection of anti-N immune responses (or other non-neutralizing antibody responses) may be related to mechanisms such as antibody-dependent cell-mediated cytotoxicity (ADCC) or complement-dependent cytotoxicity (CDC). Consequently, infected cells coated with anti-N antibodies complexed with presented N antigens or peptides, become a target for ADCC or CDC [47]. Influenza virus conserved internal nucleoprotein promotes heterosubtypic immunity due to antibodies against the NP involving CD8+T cells in an antibody dependent manner [48] and mice immunized with an ubiquitinated form of the RVFV nucleoprotein displayed higher levels of anti-N antibodies and antigen-specific T-cell responses [49]. Recent data confirmed the RVFV nucleoprotein as a potent human CD8+ T cell antigen [50].

Measurement of the viral RNA loads in blood by RT-qPCR showed that all non-viremic mice vaccinated with rMVA-Gn/Gc had higher C_t_ values than the viremic mice from the other vaccine groups (not shown), suggesting the potential of rMVA-Gn/Gc to induce close to sterile immunity. Nonetheless, other inoculation routes distinct to the ip route (eg intradermal/intramuscular) might be also tested, perhaps improving antigen presentation and allowing reduction in the virus titer needed for immunization. Therefore, one of the conclusions of this study is that the expression of the RVFV glycoprotein components by means of an attenuated poxvirus vector (MVA) is a valid strategy for development of a successful vaccine approach. Recently, other authors have shown that attenuated vaccinia viruses (VACv) expressing RVFV glycoproteins GnGc alone or in combination with human IFN-γ [51] protected CBA mice upon two vaccine administrations. However, after a single dose vaccination the survival rates were 50% and 10% respectively. In our hands, a single vaccination with rMVA-Gn/Gc was sufficient to induce higher protection levels after lethal challenge in BALB/c mice as well as in 129Sv/Ev mice. These discrepancies may be related either with the mouse strain used or the blocking of IFNα/β responses by VACv soluble receptors. In MVA the product of the B18R gene, coding for an IFN α/β binding protein is absent [52], [53], therefore functional levels of this cytokine are detected in sera from mice vaccinated with rMVA-Gn/Gc as early as 6 hours post vaccination. This difference is of particular importance since the role of IFN α/β in preventing viral infection is not limited only to the induction of an antiviral state but it has also important effects on the induction of adaptive immune responses. Type-I IFNs may exert direct effects on professional antigen presenting cells (APCs) by licensing them towards antigen processing and cross-presentation as well as through direct stimulation of CD8+T cells [30]. Direct stimulation of CD8+ T cells pre-activate this cell population to respond much faster to MHC-I restricted antigens [54]. Our data are in good agreement with these observations since impaired IFN responses (in IFNAR^−/−^ mice) completely abolished the protective effect of the vaccine. Other authors have shown a similar effect when treating immunized mice with anti IFN-α antibodies [55], therefore impairing temporarily the signaling through IFNAR. Although we have no evidence of glycoprotein specific CD8+-T cells becoming directly activated by MVA induced IFN-α/β, a subset of these cells responded specifically to glycoprotein Gn and Gc peptide sequences. It would be interesting to know whether in the absence of IFN response the peptide stimulation of CD8+T cells occurs similarly. We have shown previously that DNA vaccination with plasmid pCMV-M4 protects IFNAR^−/−^ mice from lethal RVFV challenge [36]. In contrast, the same glycoprotein antigens delivered by means of a MVA vaccine are not able to protect this strain of mice upon challenge. Therefore the vaccine delivery method used appears to greatly influence the outcome. It is noteworthy to mention that CD8+T cell responses could be more efficiently primed in IFNAR^−/−^ mice than in wt mice upon DNA vaccination [56]. Taken together this emphasizes a potential involvement for CD8+-T cells in protection against RVFV.

Finally, the induction of T-cell responses to the well-characterized CD8+ T cell peptide pb9 was consistently higher when this epitope was fused to the viral nucleoprotein rather than to the glycoprotein sequence. This fact indicates that the viral nucleoprotein is efficiently processed intracellularly either within the MVA-infected cell or after APC uptake. Moreover, N cell uptake would be favored since the N protein is found abundant in the supernatant of infected or transfected cells [40]. The fact that the response of pb9 predominates over the predicted epitopes in the N sequence may be due to the absence of other MHC-I epitopes in the N polypeptide. More work is needed to determine whether the RVFV nucleoprotein ORF carries also immune relevant MHC-I restricted epitopes. On the other hand, the glycoprotein-pb9 responses elicited were consistently lower than those of the glycoprotein specific peptides. This was somewhat unexpected since pb9 is a potent CTL stimulator. One possible explanation is that the T-cell responses towards the Gn or Gc peptides are immunodominant over the response induced by pb9 or, perhaps, the proper processing of pb9 for presentation is impaired. In any case the identified T-cell epitopes will be a very interesting tool for the rationale design of novel RVFV vaccines and to understand the role of CD8+T cells in protection.
